# Associations between green space exposure and climate anxiety among Chinese university students: the role of school-based and leisure-time physical activity, nature connectedness, and perceived stress

**DOI:** 10.3389/fpsyg.2026.1948606

**Published:** 2026-09-24

**Authors:** Xueqin Wu, Yan Liu, Cui Deng, Hamid Amin

**Affiliations:** 1 School of Wushu (Martial Arts), Wuhan Sports University, Wuhan, Hubei, China; 2 School of Environmental Science and Engineering, Huazhong University of Science and Technology, Wuhan, China; 3 Department of Psychology, College of Arts, Mustansiriyah University, Baghdad, Iraq

**Keywords:** climate anxiety, green space, nature connectedness, perceived stress, physical activity

## Abstract

**Background:**

Climate anxiety has become an important mental health concern among young adults, yet the pathways linking green space exposure to climate-related distress remain unclear. This study examined whether objective green space exposure was associated with climate anxiety among Chinese university students through domain-specific physical activity, nature connectedness, and perceived stress.

**Methods:**

A cross-sectional study included 918 undergraduate students from four universities in Wuhan Province, China. Residential and campus greenness were assessed using the satellite-derived Normalized Difference Vegetation Index (NDVI). Moderate-to-vigorous physical activity (MVPA) was objectively measured using accelerometers and classified as school-based or leisure-time MVPA. Nature connectedness, perceived stress, and climate anxiety were assessed using validated questionnaires. Structural equation modeling with bias-corrected bootstrapping (5,000 resamples) was used to estimate direct and indirect associations while adjusting for relevant covariates.

**Results:**

Residential greenness was positively associated with leisure-time MVPA and nature connectedness, whereas campus greenness was more strongly associated with school-based MVPA. No significant direct association was observed between residential greenness and climate anxiety after accounting for mediators. However, an inverse indirect association was identified through leisure-time MVPA, nature connectedness, and perceived stress. Leisure-time MVPA showed stronger associations with climate anxiety than school-based MVPA. Nature connectedness displayed a positive direct association with climate anxiety but an inverse indirect association through lower perceived stress. Perceived stress showed the strongest association with climate anxiety.

**Conclusion:**

These findings suggest that green space may be linked to climate anxiety primarily through behavioral and psychological pathways.

## 1 Introduction

Climate change is increasingly recognized as an important source of psychological distress, with climate anxiety emerging as a critical mental health concern, particularly among young adults (Seth et al., 2023). While traditional environmental psychology frameworks, such as Stress Reduction Theory and Attention Restoration Theory, posit that exposure to green spaces promotes psychological restoration and reduces stress and general psychological distress (Galera et al., 2025), climate anxiety may not be fully understood through models developed for general psychological distress. Unlike generalized emotional distress, which often centers on personal life events or immediate psychosocial stressors, climate anxiety is directed toward an abstract, large-scale, and often uncontrollable global ecological threat (Von Gal et al., 2024). Although climate anxiety shares certain emotional features with generalized anxiety and psychological distress, it represents a conceptually distinct construct because it is specifically elicited by awareness of climate change and its anticipated consequences rather than by immediate personal or psychosocial stressors (Crandon et al., 2024). Whereas generalized psychological distress reflects a broad pattern of emotional suffering arising from diverse life circumstances (Piao et al., 2024), climate anxiety encompasses cognitive-emotional responses, persistent climate-related worry, and functional impairment linked specifically to environmental change (Crandon et al., 2024).

Consequently, green environments may exert a paradoxical influence: while providing restorative psychological benefits, they might simultaneously heighten environmental awareness and distress among sensitive individuals (Kumari et al., 2025). Together, these observations suggest that existing restorative models may not adequately explain how green space relates to climate anxiety. Most previous studies have examined associations between green space exposure and depression, generalized anxiety, perceived stress, or overall psychological well-being (Liu et al., 2023; Wang et al., 2024). Whether the restorative mechanisms proposed for these broader mental health outcomes also extend to climate-specific emotional responses remains insufficiently understood. Accordingly, understanding this relationship requires attention not only to the amount of green-space exposure but also to the behavioral and psychological processes through which exposure may relate to climate-related distress.

Among the mechanisms through which green environments may influence psychological functioning, physical activity has received substantial empirical attention (Wicks et al., 2022; Li et al., 2022). However, its psychological relevance may depend on the context in which it occurs. For university students, physical activity can be distinguished between institutionally structured school-based activity and more autonomous leisure-time activity. Leisure-time activity is characterized by greater self-determination and voluntary environmental engagement, which may shape psychological responses differently from mandatory or routine physical education (Wicks et al., 2022). Reviews of greenspace research have identified physical activity and perceived stress as potential pathways linking greater greenness to mental well-being, although the strength of mediation varies across studies (Liu et al., 2024; Zare Sakhvidi et al., 2023). Studies among university students have likewise reported that active engagement with green spaces is associated with lower perceived stress and better well-being (Bai et al., 2024). On this basis, the present study distinguished school-based from leisure-time MVPA rather than treating physical activity as a homogeneous behavior. We expected residential greenness to be more closely related to leisure-time MVPA because residential environments provide opportunities for discretionary activity, whereas campus greenness was expected to show a stronger association with school-based MVPA because the campus is the primary setting for institutionally organized physical activity.

These behavioral pathways may also shape psychological responses through nature connectedness. Nature connectedness may play a dual psychological role in the context of ecological crises (Colombo et al., 2025). Greater nature connectedness has been associated with better psychological well-being and lower psychological burden (Grabowska-Chenczke et al., 2022), and systematic evidence suggests that connection with nature may be relevant to stress-related responses (Gál and Dömötör, 2023). Nature connectedness may also be strengthened through direct and active engagement with natural environments, making leisure-time activity a potentially relevant behavioral pathway (Teixeira et al., 2023). At the same time, emerging climate-anxiety research suggests that stronger nature connectedness may coexist with greater sensitivity to environmental degradation (Whitmarsh et al., 2022; Lin et al., 2025). Accordingly, the present model specified paths from residential and campus greenness to nature connectedness, as well as a pathway from leisure-time MVPA to nature connectedness, together with pathways from nature connectedness to perceived stress and climate anxiety.

Nature connectedness may therefore operate through opposing psychological processes. On one hand, a strong subjective connection to the natural world may function as a psychological resource associated with lower perceived stress (Lin et al., 2025). On the other hand, stronger connectedness may be associated with greater emotional investment in ecological systems and greater sensitivity or vulnerability when individuals are confronted with environmental degradation (Pihkala, 2022). Elevated perceived stress may itself represent a proximal vulnerability factor for climate-related distress by reducing the psychological resources available to cope with an ongoing and difficult-to-control environmental threat. Perceived stress was therefore positioned as a proximal pathway linking behavioral and environmental factors to climate anxiety. Prior research has identified stress reduction as a common mechanism connecting green-space exposure with mental well-being (Gong et al., 2024), while physical activity may also contribute to stress-related outcomes (Wicks et al., 2022). Accordingly, both school-based and leisure-time MVPA were modeled as predictors of perceived stress, alongside nature connectedness, and higher perceived stress was expected to be associated with greater climate anxiety.

Taken together, Stress Reduction Theory (SRT) and Attention Restoration Theory (ART) provide complementary perspectives for understanding the restorative potential of green environments. SRT emphasizes stress reduction and recovery, whereas ART emphasizes restoration of directed attention through restorative environmental experiences (Li et al., 2025). Building on these perspectives, the present study extends the conventional exposure–restoration framework by examining behavioral and psychological pathways through which green-space exposure may relate to climate anxiety, including domain-specific physical activity, nature connectedness, and perceived stress.

Chinese universities provide a highly relevant context for investigating these dynamics because students are simultaneously exposed to highly urbanized residential environments and deliberately planned green campus settings (Ge et al., 2025). This structural variation allows for the crucial distinction between residential and campus greenness to be examined, illuminating how different types of everyday exposure influence behavior and well-being. Recent evidence further suggests that specific characteristics of campus green spaces contribute to students’ environmental experiences in ways that differ from other daily settings, underscoring the importance of treating campus greenness as a distinct environmental exposure (Ma et al., 2025). Wuhan provides a particularly relevant setting because it is located in the middle reaches of the Yangtze River and has a humid subtropical monsoon climate characterized by hot summers and substantial rainfall (Cui et al., 2023). The broader Yangtze River Basin has experienced recurrent and increasingly severe heat extremes, including an exceptionally intense and persistent regional heatwave in summer 2022 (Feng et al., 2026). These characteristics provide a relevant real-world context for examining environmental exposure and climate-related psychological responses, while Wuhan’s urban landscape and variation in residential and campus green environments provide an appropriate setting for investigating the proposed environmental pathways.

Accordingly, the present study examined the indirect associations between objective residential and campus greenness and climate anxiety among Chinese university students. We investigated whether these associations differed by domain of physical activity (school-based versus leisure-time) and operated through nature connectedness and perceived stress. By focusing on climate anxiety as a climate-specific psychological outcome, the study extends research on green-space and mental health beyond conventional restorative outcomes. Guided by this framework, we hypothesized that residential greenness would be associated with both school-based and leisure-time MVPA, with leisure-time MVPA additionally related to nature connectedness. Campus greenness was expected to show a stronger association with school-based MVPA. Both MVPA domains and nature connectedness were expected to be associated with lower perceived stress, while nature connectedness was also modeled as a direct correlate of climate anxiety given the possibility that stronger environmental identification may increase sensitivity to climate-related threats. Perceived stress was specified as the proximal psychological correlate of climate anxiety.

## 2 Methods

### 2.1 Study design and participants

This cross-sectional study was conducted between September 2025 and January 2026 among undergraduate students enrolled at four comprehensive universities in Wuhan, Hubei Province, China. Wuhan is a major urban center in the middle reaches of the Yangtze River and has a humid subtropical monsoon climate characterized by hot, humid summers and substantial seasonal rainfall. The four campuses were situated around the East Lake (Donghu) area, one of the largest urban green spaces in Wuhan, thereby providing a range of campus greenness conditions within the same metropolitan context. A multistage cluster sampling strategy was employed to recruit participants from institutions differing in size, geographic setting, and campus environmental characteristics. Initially, participating universities were selected based on institutional agreement and the availability of geographic information necessary for objective environmental assessment. Within each university, academic departments were randomly selected, followed by the random selection of intact classes from those departments. All eligible students within selected classes were invited to participate. Mean campus NDVI values ranged from 0.38 to 0.49, indicating moderate variation in campus greenness while maintaining comparable educational settings. The geographical distribution of the participating universities is presented in Figure 1.

**Figure 1:**
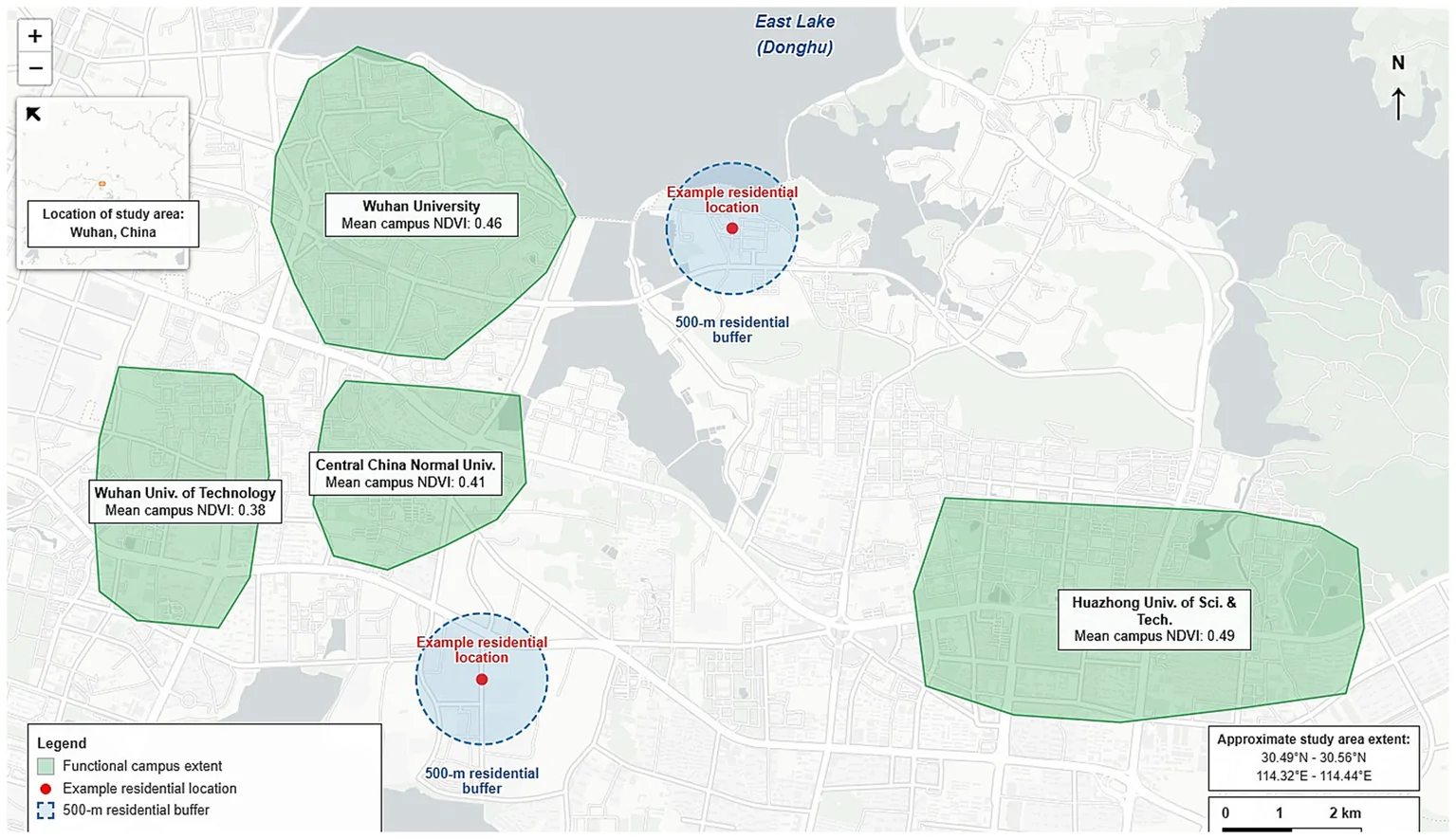
Study area in Wuhan, China. Green polygons indicate the functional campus extents used for campus-level Normalized Difference Vegetation Index (NDVI) estimation. Red points and dashed circles represent illustrative residential locations and 500-m residential buffers included solely to demonstrate the exposure assessment procedure and do not correspond to actual participant residences. The inset map shows the geographic location of the study area within Wuhan, China.

Students were considered eligible if they were full-time undergraduate students aged between 18 and 25 years, were able to read and understand Chinese, agreed to wear an accelerometer for seven consecutive days, and provided written informed consent. Students were excluded if they reported physician-diagnosed psychiatric disorders that could substantially influence questionnaire responses, had physical conditions restricting participation in regular physical activity, failed to satisfy accelerometer wear-time criteria, or were unable to provide verifiable residential address information required for geospatial analyses.

Sample size estimation was initially performed using G*Power version 3.1 under a multiple-regression framework. Assuming a small effect size (f^2^ = 0.05), an alpha level of 0.05, statistical power of 0.95, and 12 predictors, the minimum required sample size was estimated at 531 participants. Because structural equation modeling (SEM) constituted the primary analytic approach, this regression-based estimate was treated as a lower-bound planning benchmark. A larger analytical target of 900 participants was prespecified to provide adequate stability for the hypothesized SEM, based on the planned criterion of 20 observations per estimated parameter (Kline, 2023). To account for an anticipated 20 to 25% data loss related to invalid accelerometer recordings, incomplete monitoring, and geospatial geocoding failures, approximately 1,150 participants were targeted for recruitment, and 1,156 students were ultimately recruited. Following data cleaning and exclusion procedures, 918 participants remained in the final analytical sample. Thus, the final sample substantially exceeded both the GPower minimum of 531 participants and the prespecified SEM target of 900 participants.

Ethical approval for this study was obtained from the Institutional Review Board (IRB) of Huazhong University of Science and Technology, Wuhan, China (Approval No. HUST-25-X102). All procedures were conducted in accordance with the Declaration of Helsinki. Written informed consent was obtained from all participants prior to their inclusion in the study.

### 2.2 Data collection procedures

Data collection took place during supervised classroom sessions conducted by trained research assistants. Participants first completed paper-based questionnaires assessing demographic characteristics and psychological constructs. Information regarding residential addresses and university dormitory residence was subsequently obtained using standardized forms for geospatial analyses. Following questionnaire completion, participants were fitted with ActiGraph GT3X + accelerometers and provided with structured activity diaries. Written and verbal instructions regarding device wear and diary completion were provided. Participants were contacted daily through mobile messaging applications throughout the monitoring period to encourage compliance. Upon completion of the seven-day assessment period, accelerometers and activity diaries were collected by research personnel.

### 2.3 Assessment of green space exposure

Objective green space exposure was assessed using satellite-derived vegetation indices and Geographic Information Systems (GIS). Because university students spend substantial amounts of time both within residential neighborhoods and campus environments, greenness was evaluated separately for residential and campus settings (Markevych et al., 2017). Figure 1 also illustrates the spatial framework used to distinguish campus greenness from residential greenness, including examples of the 500-m residential buffers used for neighborhood greenness assessment.

Residential greenness was assessed using participants’ primary residential addresses during the academic semester. Addresses were geocoded using ArcGIS Pro (Esri Inc., Redlands, CA, USA), and all geocoding procedures were manually verified to ensure positional accuracy. Vegetation density surrounding each residence was quantified using the Normalized Difference Vegetation Index (NDVI) and the Enhanced Vegetation Index (EVI), derived from cloud-free Sentinel-2 satellite imagery corresponding to the study period. Sentinel-2 imagery with a spatial resolution of 10 meters was processed using Google Earth Engine (Gorelick et al., 2017).

Mean NDVI and EVI values were extracted within circular buffers of 300 m, 500 m, and 1,000 m surrounding each residential location. Consistent with previous environmental epidemiological research, the 500-m buffer was designated as the primary exposure metric representing the walkable neighborhood environment (Labib et al., 2020), whereas the 300-m and 1,000-m buffers were used in sensitivity analyses. Higher values indicated greater surrounding greenness (Kwan, 2018).

Campus greenness was assessed using official campus boundary shapefiles provided by the participating universities. The boundary datasets were visually verified against recent high-resolution satellite imagery to ensure spatial consistency with the actual campus extents prior to analysis. Mean NDVI and EVI values were subsequently calculated for each campus polygon using the same Sentinel-2 imagery and preprocessing procedures applied to the residential exposure assessment. Residential and campus greenness indicators were entered simultaneously into the analytical models to estimate their independent associations with climate anxiety. This approach enabled the independent contributions of residential and campus greenness to be estimated while accounting for their shared variance.

### 2.4 Sociodemographic and anthropometric variables

Participants completed a brief demographic questionnaire that collected information on age, sex, year of study, urban or rural background, only-child status, and subjective socioeconomic status. Subjective socioeconomic status was assessed using the MacArthur Scale of Subjective Social Status, in which participants indicate their perceived social standing relative to others within Chinese society (Li et al., 2024). Height and body weight were self-reported, and body mass index (BMI) was subsequently calculated as weight in kilograms divided by height in meters squared. These variables were included as covariates because of their documented associations with physical activity participation and mental health outcomes. These characteristics were prespecified as potential confounders because demographic, socioeconomic, family-structure, and residential-background factors may be associated with physical activity patterns and psychological well-being. In particular, only-child status was considered because previous research among Chinese students has reported associations between only-child status and perceived stress (Liu et al., 2025), whereas urban or rural background may capture differences in socioeconomic and environmental context, health behaviors, and psychological well-being (Tuersun et al., 2025). Covariate selection was prespecified on theoretical and empirical grounds and was not based on statistical significance in the present sample.

### 2.5 Objective assessment of physical activity

Physical activity was objectively measured using the ActiGraph GT3X + accelerometer (ActiGraph LLC, Pensacola, FL, USA), a research-grade device widely used in public health research (Migueles et al., 2017). Participants were instructed to wear the monitor on an elastic belt positioned over the right hip during waking hours for seven consecutive days, removing it only during sleep, bathing, swimming, or other water-based activities. Accelerometer data were downloaded and processed using ActiLife software version 6. Data were recorded using 60-s epochs. Non-wear time was defined as at least 60 consecutive minutes of zero activity counts, allowing for up to 2 min of interruption. A valid monitoring day required at least 10 h of wear time, and a valid assessment period required a minimum of four valid weekdays and one valid weekend day (Gao et al., 2022).

Activity intensity categories were classified using Freedson adult cut-points applied to vertical axis counts (Migueles et al., 2017; Freedson et al., 1998). Sedentary behavior was defined as fewer than 100 counts per minute, light-intensity activity as 100–1951 counts per minute, moderate activity as 1952–5,724 counts per minute, and vigorous activity as 5,725 counts per minute or greater. Moderate-to-vigorous physical activity (MVPA) was defined as all activity accumulated at or above 1952 counts per minute. Weekly minutes of MVPA were used as the primary indicators of physical activity.

### 2.6 Classification of physical activity domains

Because accelerometers do not provide contextual information regarding the setting in which activity occurs, participants completed structured activity diaries during the seven-day monitoring period. The diaries recorded the timing, duration, and contextual description of physical education classes, organized university sports activities, scheduled campus exercise sessions, and recreational activities undertaken outside institutional obligations. Diary records were temporally matched to accelerometer-derived MVPA periods to determine the context in which each MVPA episode occurred. To improve the reliability of domain classification, two trained researchers independently coded the activity records using a prespecified coding protocol developed before the final classification. MVPA episodes occurring during scheduled or institutionally organized activities were classified as school-based physical activity, whereas MVPA occurring outside institutional obligations and representing self-directed or recreational activity was classified as leisure-time physical activity. Records with incomplete timing information or insufficient contextual information were flagged as ambiguous rather than being assigned to a domain based on researcher judgment alone. Ambiguous records were reviewed using the available diary information and, where applicable, corresponding institutional schedules; records that remained unresolved were adjudicated by (a third trained researcher). Inter-coder agreement for the categorical domain classification was high (Cohen’s *κ* = 0.84; percentage agreement = 92.8%) before discrepancies were resolved. The final domain classification was subsequently used to calculate weekly school-based and leisure-time MVPA.

### 2.7 Nature connectedness

Nature connectedness was assessed using the validated Chinese version of the Nature Relatedness Scale (NRS-C) (Zuo et al., 2025). The NRS-C is a 17-item multidimensional instrument specifically adapted for Chinese university students, comprising three distinct subscales: NR-self, NR-perspective, and NR-experience. Items were rated on a standard 5-point Likert scale ranging from 1 (strongly disagree) to 5 (strongly agree). A composite score was calculated by averaging the responses across all 17 items, with higher scores indicating a stronger subjective connection to the natural environment. In the present study, the scale exhibited acceptable reliability (Cronbach’s *α* = 0.71).

### 2.8 Perceived stress

Perceived stress was measured using the Chinese version of the 10-item Perceived Stress Scale (PSS-10) (Du et al., 2023). The PSS-10 assesses the extent to which individuals perceive situations in their lives as stressful during the preceding month. Items are rated on a 5-point scale ranging from 0 (never) to 4 (very often). Four positively worded items are reverse-coded prior to scoring. Total scores range from 0 to 40, with higher scores reflecting greater perceived stress. The Chinese version of the PSS-10 has demonstrated good internal consistency and construct validity among Chinese university students (Du et al., 2023). In the current sample, the scale yielded an adequate reliability coefficient (Cronbach’s *α* = 0.74).

### 2.9 Climate anxiety

Climate anxiety was assessed using the Climate Anxiety Scale (CAS) developed by Clayton and Karazsia (2020). The CAS comprises 13 items evaluating cognitive-emotional impairment and functional impairment related to climate change concerns. Participants indicated how frequently they experienced each reaction using a 5-point Likert scale ranging from 1 (never) to 5 (almost always). Mean scores across all items were calculated, with higher values indicating greater levels of climate-related anxiety. Previous research conducted among Chinese young adults has reported acceptable psychometric properties for the Chinese version of the CAS (Lau et al., 2025). For the present sample, the instrument showed an acceptable level of internal reliability (Cronbach’s α = 0.70).

### 2.10 Data quality control

All research assistants completed standardized training prior to participant recruitment and data collection. Completed questionnaires were reviewed immediately after administration to identify missing responses whenever possible. Double data entry procedures were employed to minimize transcription errors. Accelerometer data processing was independently performed by two members of the research team, and discrepancies were resolved through discussion. Geocoded residential locations and campus boundaries were visually inspected using satellite imagery to ensure spatial accuracy.

### 2.11 Statistical analysis

Statistical analyses were conducted using SPSS version 29.0 and AMOS version 29.0. Descriptive statistics were calculated for all study variables, and internal consistency was assessed using Cronbach’s alpha coefficients. Missing data patterns were examined using Little’s MCAR test. As the missingness was consistent with the assumption of missing completely at random, Full Information Maximum Likelihood (FIML) estimation was used in subsequent structural analyses. Pearson correlation analyses were first conducted to describe the unadjusted bivariate associations among environmental, behavioral, and psychological variables and to provide an initial overview of the relationships among study constructs. These correlations were descriptive and did not account simultaneously for covariates, competing predictors, or the hypothesized mediation pathways.

Structural equation modeling (SEM) was subsequently used to test the prespecified multivariable structural model, allowing the direct and indirect associations among the study variables to be estimated simultaneously while adjusting for relevant covariates. Age, sex, body mass index, subjective socioeconomic status, year of study, and urban or rural background were included as covariates. Indirect effects were tested using bias-corrected bootstrap procedures with 5,000 resamples, and statistical significance was determined when 95% confidence intervals did not include zero.

Sensitivity analyses were conducted to assess the robustness of the findings using an alternative greenness indicator (EVI), residential buffer sizes of 300 m and 1,000 m, and alternative model specifications. Multi-group SEM analyses were also performed to assess potential differences in the structural associations between male and female participants. Model fit was evaluated using the chi-square statistic, Comparative Fit Index (CFI), Tucker–Lewis Index (TLI), Root Mean Square Error of Approximation (RMSEA), and Standardized Root Mean Square Residual (SRMR). Values of CFI and TLI ≥ 0.95, RMSEA ≤ 0.06, and SRMR ≤ 0.08 were considered indicative of good model fit.

## 3 Results

A total of 1,156 undergraduate students from four universities were initially recruited. Of these, 67 students declined to wear the accelerometer after enrollment, 54 returned devices with technical failures or incomplete monitoring periods, and 78 did not meet the predefined accelerometer wear-time criteria. In addition, 39 participants were excluded because residential addresses could not be accurately geocoded, precluding objective assessment of residential greenness. After data cleaning procedures, 918 participants were retained in the final analytical sample, corresponding to an overall retention rate of 79.4%.

The final analytical sample comprised 918 participants (mean age = 20.3 ± 1.5 years), of whom 56.1% were female. Most participants were in their second year of study (31.3%), 58.2% reported an urban background, and 44.7% were only children. The mean BMI was 22.0 ± 3.1 kg/m^2^. Descriptive statistics for environmental exposure measures, physical activity variables, and psychological constructs are presented in Table 1.

**Table 1:** Participant characteristics, descriptive statistics, and reliability estimates of study variables (N = 918).

Characteristic		*n* (%) or Mean ± SD
Age (years)		20.3 ± 1.5
Sex	Female	515 (56.1%)
	Male	403 (43.9%)
BMI (kg/m^2^)		22.0 ± 3.1
Year of study	Year 1	216 (23.5%)
	Year 2	287 (31.3%)
	Year 3	243 (26.5%)
	Year 4	172 (18.7%)
Background	Urban background	534 (58.2%)
	Rural background	384 (41.8%)
Family status	Only child	410 (44.7%)
	Non-only child	508 (55.3%)
Subjective socioeconomic status	Low to lower-middle	140 (15.2%)
	Middle	628 (68.4%)
	Upper-middle to high	150 (16.4%)
Residential NDVI (500 m)		0.41 ± 0.11
Residential EVI (500 m)		0.30 ± 0.08
Campus NDVI		0.47 ± 0.09
Campus EVI		0.35 ± 0.07
School-based MVPA (min/week)		92.4 ± 35.6
Leisure-time MVPA (min/week)		66.2 ± 39.7
Total MVPA (min/week)		158.6 ± 61.4
Nature relatedness (NRS-C)		3.54 ± 0.63
Perceived stress (PSS-10)		17.9 ± 5.9
Climate anxiety (CAS)		2.61 ± 0.72

NDVI, Normalized Difference Vegetation Index; EVI, Enhanced Vegetation Index; MVPA, Moderate-to-Vigorous Physical Activity; NRS, Nature Relatedness Scale; PSS-10, Perceived Stress Scale; CAS, Climate Anxiety Scale.

Missing questionnaire responses were minimal (<3.5% across all items). Little’s MCAR test was not statistically significant (χ^2^ = 51.84, df = 43, *p* = 0.17), indicating that the missing data were consistent with the assumption of missing completely at random. Accordingly, Full Information Maximum Likelihood estimation was used in subsequent structural analyses.

Pearson correlation coefficients among study variables are presented in Table 2. Residential NDVI was positively correlated with leisure-time MVPA (r = 0.29, *p* < 0.001), nature connectedness (r = 0.24, *p* < 0.001), and campus NDVI (r = 0.18, *p* < 0.001). Associations with school-based MVPA were comparatively weaker (r = 0.12, *p* < 0.001).

**Table 2:** Unadjusted Pearson correlation coefficients among environmental, behavioral, and psychological study variables.

Variable	Residential NDVI	Campus NDVI	School-based MVPA	Leisure-time MVPA	Nature connectedness	Perceived stress	Climate anxiety
Residential NDVI	–						
Campus NDVI	0.18^***^	–					
School-based MVPA	0.12^***^	0.21^***^	–				
Leisure-time MVPA	0.29^***^	0.17^***^	0.11^**^	–			
Nature connectedness	0.24^***^	0.19^***^	0.09^**^	0.34^***^	–		
Perceived stress	−0.19^***^	−0.16^***^	−0.13^***^	−0.20^***^	−0.31^***^	–	
Climate anxiety	−0.10^**^	−0.08^*^	−0.06	−0.11^**^	0.09^**^	0.44^***^	–

^*^p < 0.05; ^**^p < 0.01; ^***^p < 0.001.

Campus NDVI demonstrated modest positive correlations with school-based MVPA (r = 0.21, p < 0.001), leisure-time MVPA (r = 0.17, *p* < 0.001), and nature connectedness (r = 0.19, *p* < 0.001). Both residential and campus greenness were inversely associated with perceived stress and climate anxiety, although the magnitude of these relationships was generally small.

Nature connectedness was negatively associated with perceived stress (r = −0.31, p < 0.001) but showed a weak positive correlation with climate anxiety (r = 0.09, *p* = 0.008). Perceived stress demonstrated the strongest association with climate anxiety (r = 0.44, *p* < 0.001). Leisure-time MVPA was inversely associated with both perceived stress (r = −0.20, *p* < 0.001) and climate anxiety (r = −0.11, *p* = 0.001), whereas the corresponding correlations for school-based MVPA were smaller in magnitude.

The Pearson correlation analysis provided an initial description of the unadjusted pairwise relationships among the study variables. These associations were not intended to represent the structural pathways tested in the hypothesized model because they did not account simultaneously for covariates or the interdependence among multiple predictors and mediators. SEM was therefore used as the primary multivariable analysis to determine whether the observed associations persisted after adjustment and to quantify the direct and indirect pathways specified *a priori*.

The final structural model demonstrated excellent fit to the data (χ^2^/df = 1.95, CFI = 0.96, TLI = 0.95, RMSEA = 0.04 [95% CI: 0.03–0.05], SRMR = 0.03) and explained 18.2% of the variance in nature connectedness, 27.4% of the variance in perceived stress, and 31.6% of the variance in climate anxiety. The final structural model, standardized path coefficients, and explained variance are presented in Figure 2.

**Figure 2:**
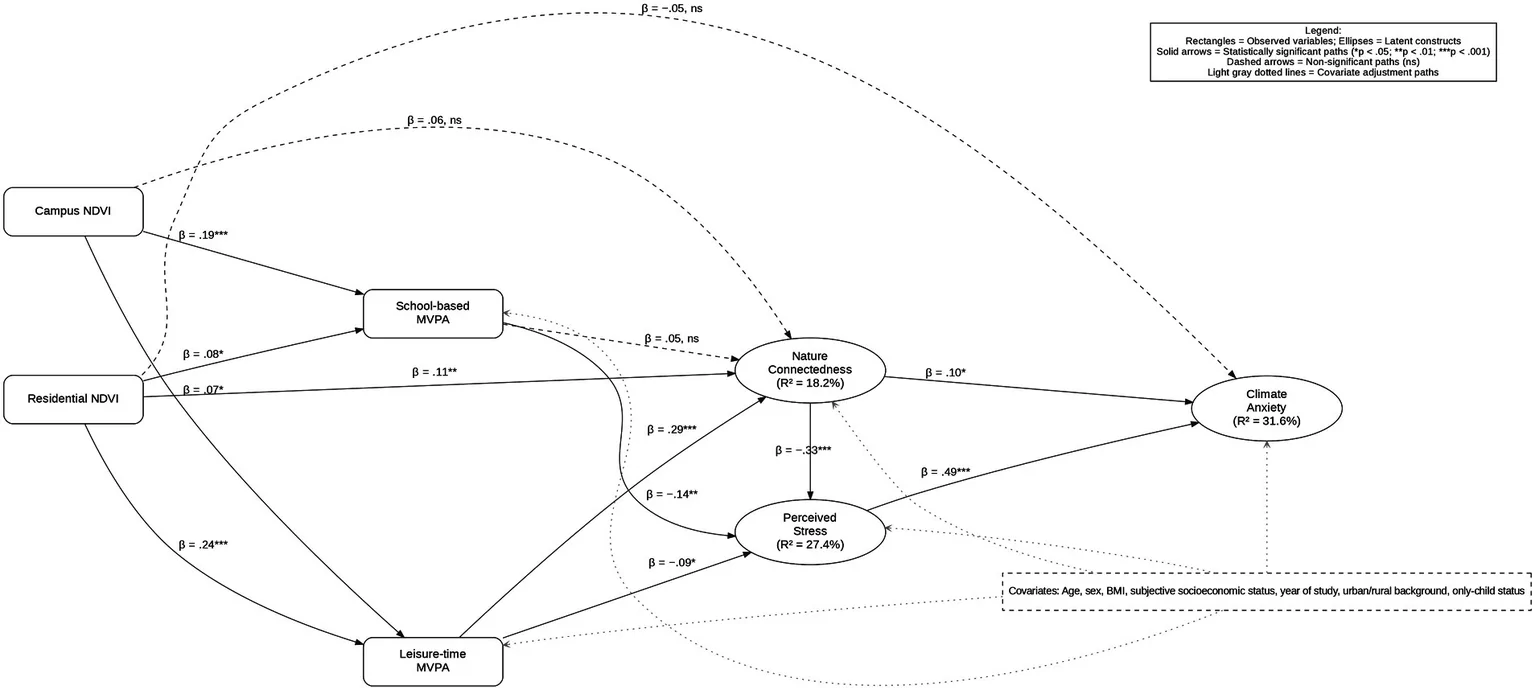
Final structural equation model with standardized path coefficients. Solid arrows indicate statistically significant structural paths, whereas dashed arrows indicate non-significant structural paths. Light gray dotted lines indicate covariate adjustment paths. Values shown on arrows are standardized regression coefficients (β). *R*^2^ values indicate the proportion of variance explained in each endogenous variable. NDVI, Normalized Difference Vegetation Index; MVPA, moderate-to-vigorous physical activity; ns, non-significant. *p < 0.05; **p < 0.01; ***p < 0.001.

Residential NDVI was positively associated with school-based MVPA (*β* = 0.08, *p* = 0.032), leisure-time MVPA (β = 0.24, *p* < 0.001), and nature connectedness (β = 0.11, *p* = 0.004). The direct association between residential NDVI and climate anxiety was not statistically significant after accounting for the remaining variables in the model (*β* = −0.05, *p* = 0.181). Campus NDVI was positively associated with school-based MVPA (β = 0.19, p < 0.001) and showed a weaker association with leisure-time MVPA (β = 0.07, *p* = 0.041). The direct effect of campus NDVI on nature connectedness was not statistically significant (*β* = 0.06, *p* = 0.087).

Regarding behavioral pathways, leisure-time MVPA was positively associated with nature connectedness (β = 0.29, *p* < 0.001) and inversely associated with perceived stress (*β* = −0.09, *p* = 0.018). In contrast, school-based MVPA was not significantly associated with nature connectedness (*β* = 0.05, *p* = 0.148) but demonstrated a direct inverse association with perceived stress (*β* = −0.14, *p* = 0.002).

For the psychological constructs, nature connectedness was inversely associated with perceived stress (*β* = −0.33, *p* < 0.001). However, a small positive direct association was observed between nature connectedness and climate anxiety (*β* = 0.10, *p* = 0.011). Perceived stress exhibited the strongest direct association with climate anxiety (*β* = 0.49, *p* < 0.001). Standardized direct, indirect, and total effects from the final structural model are summarized in Table 3.

**Table 3:** Standardized direct, indirect, and total effects from the final structural model (*N* = 918).

Pathway		Direct effect (β)	*p*-value (Direct)	Indirect effect (β)	95% CI (indirect)	Total effect (β)	*p*-value (Total)
Environmental exposures	Residential NDVI → school-based MVPA	0.08	0.032	–	–	0.08	0.032
	Residential NDVI → leisure-time MVPA	0.24	< 0.001	–	–	0.24	< 0.001
	Residential NDVI → nature connectedness	0.11	0.004	0.07	[0.03, 0.11]	0.18	< 0.01
	Residential NDVI → climate anxiety	−0.05	0.181	−0.07	[−0.11, −0.03]	−0.12	0.013
	Campus NDVI → school-based MVPA	0.19	< 0.001	–	–	0.19	< 0.001
	Campus NDVI → leisure-time MVPA	0.07	0.041	–	–	0.07	0.041
	Campus NDVI → climate anxiety	–	–	−0.02	[−0.04, −0.01]	−0.06	0.074
	Campus NDVI → nature connectedness	0.06	0.087	–	–	0.06	0.087
Behavioral pathways (MVPA)	School-based MVPA →nature connectedness	0.05	0.148	–	–	0.05	0.148
	School-based MVPA → perceived stress	−0.14	0.002	–	–	−0.14	0.002
	School-based MVPA → climate anxiety	–	–	−0.07	[−0.11,-0.03]	−0.07	0.048
	Leisure-time MVPA→ nature connectedness	0.29	< 0.001	–	–	0.29	< 0.001
	Leisure-time MVPA → perceived stress	−0.09	0.018	−0.10	[−0.15, −0.06]	−0.19	< 0.001
	Leisure-time MVPA → climate anxiety	–	–	−0.12	[−0.18, −0.07]	−0.12	0.008
Psychological pathways	Nature connectedness → perceived stress	−0.33	< 0.001	–	–	−0.33	< 0.001
	Nature connectedness → climate anxiety	0.10	0.011	−0.16	[−0.21, −0.11]	−0.06	0.118
	Perceived stress → climate anxiety	0.49	< 0.001	–	–	0.49	< 0.001

*N* = 918. CI, Confidence Interval; MVPA, Moderate-to-Vigorous Physical Activity; NDVI, Normalized Difference Vegetation Index. Indirect effects were tested using bias-corrected bootstrapping with 5,000 resamples. An indirect effect is considered statistically significant if the 95% CI does not include zero. Dashes indicate that no corresponding direct or indirect effect was estimated for reporting purposes because the pathway was not specified as a focal effect in the final model.

Residential NDVI demonstrated three statistically significant indirect pathways to climate anxiety. The most conceptually comprehensive pathway operated sequentially through leisure-time MVPA, nature connectedness, and perceived stress (*β* = −0.03, 95% CI: −0.05 to −0.01), indicating that greener residential environments were associated with greater leisure-time MVPA, which was in turn associated with stronger nature connectedness and lower perceived stress, ultimately corresponding to lower climate anxiety. This full sequential pathway accounted for a substantial component of the overall indirect association between residential greenness and climate anxiety (total indirect effect *β* = −0.07, 95% CI: −0.11 to −0.03). Two additional significant indirect pathways were observed through leisure-time MVPA and perceived stress (*β* = −0.02, 95% CI: −0.04 to −0.01) and through nature connectedness and perceived stress (*β* = −0.02, 95% CI: −0.04 to −0.01).

Campus NDVI also showed a significant inverse indirect association with climate anxiety through school-based MVPA and perceived stress (β = −0.02, 95% CI: −0.04 to −0.01), although this association was weaker than the corresponding residential pathway. The indirect effects further highlighted the central role of perceived stress in the association between nature connectedness and climate anxiety. Nature connectedness demonstrated two opposing patterns of association with climate anxiety. Its indirect association through reduced perceived stress was negative (*β* = −0.16, 95% CI: −0.21 to −0.11), which contrasted with its positive direct association. Residential NDVI also demonstrated a positive indirect association with nature connectedness through leisure-time MVPA (*β* = 0.07, 95% CI: 0.03 to 0.11). Residential NDVI retained a modest inverse total association with climate anxiety (*β* = −0.12, *p* = 0.013). In contrast, the overall total association between campus greenness and climate anxiety did not reach conventional levels of statistical significance (*β* = −0.06, *p* = 0.074).

Leisure-time MVPA demonstrated an inverse total association with climate anxiety (*β* = −0.12, *p* = 0.008), whereas the total effect of school-based MVPA was weaker (*β* = −0.07, *p* = 0.048). Finally, although nature connectedness was positively associated with climate anxiety directly, its negative indirect association through perceived stress resulted in an attenuated and non-significant total effect (*β* = −0.06, *p* = 0.118).

Following measurement invariance testing, multi-group structural equation modeling was performed to examine whether the structural associations differed between male and female participants. The unconstrained model demonstrated acceptable fit (χ^2^ = 1,187.54, df = 692, CFI = 0.941, RMSEA = 0.040, SRMR = 0.051). Constraining all structural paths to equality across sex resulted in a modest reduction in model fit (ΔCFI = 0.012), indicating partial structural non-invariance.

Comparison of individual pathways identified two statistically significant sex differences. The association between leisure-time MVPA and nature connectedness was stronger among women (*β* = 0.36, *p* < 0.001) than among men (*β* = 0.21, *p* = 0.003; p for difference = 0.021). Likewise, the direct association between nature connectedness and climate anxiety was significant among women (*β* = 0.15, *p* = 0.006) but not among men (*β* = 0.04, *p* = 0.412; p for difference = 0.034). No significant sex difference was observed for the association between perceived stress and climate anxiety.

Two alternative models were estimated to examine the robustness of the hypothesized structural model. The first alternative model specified climate anxiety as a predictor of perceived stress and physical activity, thereby testing a different directional specification of the structural relationships. This model demonstrated acceptable fit (CFI = 0.918, RMSEA = 0.051) but fit the data less well than the hypothesized model, which showed CFI = 0.96 and RMSEA = 0.04.

The second alternative model omitted the direct path from nature connectedness to climate anxiety and also showed poorer fit than the hypothesized model (ΔCFI = 0.014; ΔRMSEA = 0.004). These comparisons provided support for retaining the hypothesized model as the better-fitting specification among the models examined, while not establishing temporal or causal ordering because of the cross-sectional study design.

Sensitivity analyses indicated that the main findings were robust across alternative model specifications. Replacing NDVI with EVI produced a similar pattern of associations, although the effect sizes were slightly attenuated. Analyses using 300-m and 1,000-m residential buffers also yielded results broadly consistent with those obtained using the primary 500-m buffer. The association between residential greenness and leisure-time MVPA was somewhat stronger with the 300-m buffer, whereas associations involving campus greenness remained relatively stable across buffer definitions. Across all sensitivity analyses, perceived stress remained the strongest correlate of climate anxiety. Detailed results are presented in Supplementary Table S1.

## 4 Discussion

The present study sought to contribute to environmental psychology literature by examining whether objective exposure to green environments was associated with climate anxiety among Chinese university students through domain-specific physical activity, nature connectedness, and perceived stress. Contrary to the assumption of a simple direct protective effect of greenness, residential greenness was no longer directly associated with climate anxiety after accounting for the proposed mediating pathways. Instead, the observed relationships operated predominantly through indirect behavioral and psychological mechanisms. These findings suggest that the mental health relevance of everyday green environments may not be adequately characterized by physical exposure alone. Previous studies indicate that associations between green space and mental health can be partly explained by behavioral and psychosocial pathways, including leisure-time physical activity, social cohesion, loneliness, and stress-related processes, while characteristics of green-space quality may also contribute beyond the quantity of vegetation (Wang et al., 2019; Van den Berg et al., 2019; Nguyen et al., 2021).

This interpretation is consistent with previous mediation research suggesting that environmental exposure measures do not capture the full set of processes through which green environments may relate to mental health. Wang et al. (2019) reported that physical activity, social cohesion, and other environmental pathways partly accounted for associations between urban greenery and mental well-being, with the specific mediators differing according to the greenery measure used. Similarly, Van den Berg et al. (2019) found that leisure-time physical activity and loneliness significantly mediated associations between time spent visiting green spaces and mental health. In addition, a systematic review of green-space quality indicated that specific environmental characteristics and qualities may be relevant to health outcomes beyond the mere presence or quantity of green space (Nguyen et al., 2021). Taken together, this literature does not suggest that physical exposure is unimportant; rather, it indicates that vegetation quantity or density represents only one component of a broader environment–health relationship.

These findings highlight a critical boundary condition for classical environmental psychology theories. Previous studies have consistently reported beneficial associations between green space exposure and lower levels of depression, generalized anxiety, and perceived stress (Bratman et al., 2019), often interpreted through Stress Reduction Theory (SRT) and Attention Restoration Theory (ART) (Wang et al., 2025). However, because climate anxiety reflects responses to a large-scale, future-oriented, and uncontrollable ecological threat, models of general psychological restoration may not be fully applicable to climate-specific distress. The present findings extend existing literature by demonstrating that the relationship between greenness and climate-related distress is statistically embedded within interconnected behavioral and psychological processes, underscoring the need for more nuanced theoretical frameworks.

An additional contribution of the present study is the distinction between the contexts in which physical activity occurs. Our findings suggest that the psychological relevance of green environments may be influenced not only by the amount of physical activity undertaken but also by the degree of choice, enjoyment, and self-directed engagement associated with that activity. By distinguishing between school-based and leisure-time physical activity, the structural model revealed distinct psychological pathways. Although both forms of activity were associated with lower perceived stress (White et al., 2024), only leisure-time MVPA demonstrated a meaningful positive association with nature connectedness and a significant inverse total association with climate anxiety.

Previous research on green exercise has highlighted autonomy and enjoyment as important motivational features of participation, while research comparing nature-, gym-, and sport-based exercise has identified distinct motives for exercising in natural environments (Wicks et al., 2022; Fraser et al., 2019). In parallel, mediation research has identified leisure-time physical activity as one pathway linking green-space engagement with mental health (Van den Berg et al., 2019). Together, these findings provide a plausible contextual interpretation of our observation that leisure-time MVPA was more strongly related to nature connectedness and climate anxiety than school-based MVPA. However, these studies do not establish that autonomy itself mediates the effects of green-space exposure on climate-related distress; rather, they support the broader interpretation that how individuals choose to engage with natural environments may be relevant to the psychological consequences of that engagement.

An important contribution of the present study is the pattern observed for campus greenness and school-based physical activity, which differed from the pathway identified for residential greenness and leisure-time activity. Campus greenness was positively associated with school-based MVPA, yet its total association with climate anxiety did not reach conventional statistical significance. Similarly, school-based MVPA was inversely associated with perceived stress but was not significantly associated with nature connectedness. Together, these findings suggest that institutional exposure to green environments and institutionally structured physical activity may not automatically translate into the forms of psychological engagement with nature that characterized the leisure-time pathway. One possible explanation is that university-based activity is more constrained by schedules, institutional requirements, and externally defined goals, which may limit opportunities for self-directed and personally meaningful interaction with natural environments (He and Ni, 2025). By contrast, leisure-time activity allows greater choice over the timing, setting, intensity, and purpose of participation, potentially creating more opportunities for experiential engagement with nature (Dodge et al., 2022). Importantly, autonomy was not directly measured in the present study; therefore, this interpretation should be regarded as a theoretically informed explanation rather than a demonstrated mechanism. The non-significant campus and school-based pathways should not be interpreted as evidence that campus green space or school-based physical activity lacks psychological value, but rather as indicating that their psychological relevance may depend on how much voluntary and personally meaningful engagement the environment affords.

These findings also raise an important cross-cultural question regarding the extent to which the observed pattern may vary across university environments with different norms and institutional structures surrounding physical activity. University physical education is not organized uniformly across countries, and the degree of autonomy support provided within physical education may influence students’ motivation and their engagement in physical activity beyond formal classes. Evidence from Chinese college students indicates that autonomy-supportive physical education can promote autonomous motivation toward leisure-time physical activity (Guo et al., 2026), while broader self-determination research similarly identifies autonomy support and autonomous motivation as relevant to exercise behavior (Mossman et al., 2024). Accordingly, the present findings should not be interpreted as implying that the same relative pattern between school-based and leisure-time activity would necessarily occur in all cultural settings. In university contexts where students have greater choice over physical activity, greater flexibility in how campus green spaces are used, and more autonomy-supportive physical education practices, the psychological pathways linking green-space exposure to nature connectedness and climate-related distress could plausibly be stronger. Conversely, highly structured campus environments may show a pattern more similar to that observed here. These possibilities remain empirical questions and should be tested directly through cross-cultural studies that assess autonomy-supportive climate, activity context, and cultural differences simultaneously.

Furthermore, the findings suggest that nature connectedness may play a complex, dual role in the context of climate change. Stronger nature connectedness was associated with lower perceived stress, which in turn was linked to lower climate anxiety. Simultaneously, however, nature connectedness demonstrated a small positive direct association with climate anxiety. Consequently, the overall total association between nature connectedness and climate anxiety was attenuated and no longer statistically significant. This pattern indicates that the association between nature connectedness and climate anxiety may depend on the psychological pathway through which nature connectedness operates. In particular, the findings suggest that the stress-buffering function of nature connectedness may coexist with greater sensitivity to climate-related threats, producing opposing indirect and direct associations that attenuate the overall association (Cunsolo and Ellis, 2018; Pihkala, 2020). Future longitudinal and experimental research could test whether these opposing pathways reflect stable individual differences in climate-related sensitivity or instead depend on the situational context in which people engage with and interpret natural environments.

Crucially, the structural model demonstrated that these mechanisms do not operate independently; rather, they form an interconnected sequential pathway. Leisure-time physical activity in greener residential environments fosters stronger nature connectedness, which subsequently acts as a psychological buffer by reducing perceived stress, thereby indirectly alleviating climate anxiety. Within this proposed model, perceived stress was the strongest psychological correlate of climate anxiety. This observation aligns with broader resource-based psychological literature, indicating that individuals experiencing elevated levels of general stress may possess depleted cognitive and emotional resources to cope with climate-related concerns (Hobfoll, 1989; Ojala et al., 2021). Importantly, the stress-buffering role of both domain-specific physical activity and nature connectedness further positions perceived stress as a central psychological mechanism linking environmental exposures to climate anxiety (Pasanen et al., 2014).

Practically, these findings suggest that simply increasing vegetation coverage on campuses is insufficient for addressing climate-related distress. Rather, universities and urban planners should prioritize the design of green spaces that actively facilitate voluntary, leisure-based outdoor engagement and foster meaningful connections with nature. Designing environments that encourage autonomous interaction—such as integrating unstructured nature trails, providing flexible recreational green spaces rather than purely formalized sports fields, and supporting programs for mindful nature engagement—may better support stress reduction and adaptive responses to environmental concerns. Such approaches move beyond conventional mental health initiatives by embedding behavioral autonomy and ecological attachment into student well-being strategies.

Several limitations should be acknowledged. First, the cross-sectional design precludes conclusions regarding temporal ordering or causality; all relationships must be interpreted strictly as associations. Longitudinal and experimental studies are required to establish whether the observed pathways reflect causal sequences. Second, although satellite-derived greenness indices provide objective measures of vegetation density, they do not capture qualitative characteristics of green environments, such as biodiversity, perceived safety, or aesthetic appeal. Third, despite the objective assessment of physical activity using accelerometry, the classification of activity domains relied on participant diaries and may be susceptible to contextual misclassification. Fourth, the sample consisted exclusively of university students from one Chinese province, necessitating future research to evaluate the generalizability of the proposed theoretical model in other cultural contexts. Fifth, the study did not assess several potentially relevant individual-level factors that may contribute to the observed associations. Personality characteristics, such as neuroticism and openness to experience, as well as climate-change knowledge or climate literacy, may be related to nature connectedness, perceived stress, and climate anxiety and could therefore represent potential sources of unmeasured confounding. Their omission from the structural model may have resulted in residual confounding of some direct and indirect associations, although the direction and magnitude of any resulting bias cannot be determined from the present data. Future studies should incorporate relevant personality characteristics, climate-change knowledge, and other potentially important individual-level factors to further test the robustness of the proposed pathways.

Despite these limitations, the present study contributes to the emerging literature on climate-related mental health by integrating objective environmental indicators, device-based physical activity assessment, and theoretically informed psychological mechanisms. By demonstrating that the association between greenness and climate anxiety is statistically embedded within interconnected behavioral and psychological pathways, the findings extend classical restorative models and offer a more nuanced theoretical understanding of how everyday environments relate to emotional responses to climate change.

## 5 Conclusion

In conclusion, objective exposure to greener environments was associated with lower climate anxiety among Chinese university students primarily through interconnected behavioral and psychological pathways, challenging models that assume direct environmental effects. Leisure-time physical activity emerged as a particularly important correlate, linking residential greenness with stronger nature connectedness and lower perceived stress, whereas school-based physical activity demonstrated associations predominantly through stress-related pathways. In contrast, the weaker and non-significant associations involving campus greenness and school-based physical activity highlight the potential importance of autonomy and contextual engagement in shaping the psychological relevance of green environments. Nature connectedness displayed a dual pattern of association, simultaneously reflecting heightened environmental concern and stress-buffering potential. Perceived stress, in turn, was identified as the most influential psychological correlate of climate anxiety within the structural model.

Together, these findings contribute to a more nuanced theoretical understanding of climate anxiety by demonstrating the necessity of autonomous environmental engagement and interconnected psychological mediation. Supporting student well-being in the context of increasing ecological uncertainty requires approaches that extend beyond mere environmental exposure or conventional mental health interventions. Designing spaces that actively facilitate meaningful, voluntary engagement with nature and encourage autonomous forms of physical activity represents a promising component of comprehensive strategies aimed at mitigating climate-related distress among young adults. Future longitudinal and cross-cultural studies are needed to clarify temporal relationships, examine whether the relative importance of school-based and leisure-time pathways varies across university contexts, and determine whether autonomy-supportive environments modify the psychological pathways linking green-space exposure to climate-related distress.

## Data Availability

The raw data supporting the conclusions of this article will be made available by the authors, without undue reservation.
