# Supplementary material for: Associations between green space exposure and climate anxiety among Chinese university students: the role of school-based and leisure-time physical activity, nature connectedness, and perceived stress
Source: Front Psychol. 2026 Sep 24;17:1948606. doi: 10.3389/fpsyg.2026.1948606 (PMC13646157; doi:10.3389/fpsyg.2026.1948606)
Supplement: Supplementary file 1 [file Table_1.docx]

**Supplementary Table S1. Sensitivity analyses of selected standardized direct effects and model fit across alternative greenness specifications (N = 918).**

| Structural Pathway | Primary Model (NDVI 500-m),  β (p-value) | Sensitivity 1 (EVI 500-m),  β (p-value) | Sensitivity 2 (NDVI 300-m),  β (p-value) | Sensitivity 3 (NDVI 1,000-m),  β (p-value) |
| --- | --- | --- | --- | --- |
| Residential Greenness → Leisure-time MVPA | .24 (< .001) | .21 (< .001) | .29 (< .001) | .20 (< .001) |
| Residential Greenness → School-based MVPA | .08 (.032) | .06 (.045) | .07 (.038) | .09 (.021) |
| Residential Greenness → Nature Connectedness | .11 (.004) | .09 (.011) | .12 (.002) | .09 (.015) |
| Campus Greenness → School-based MVPA | .19 (< .001) | .17 (.002) | .19 (< .001) | .18 (< .001) |
| Campus Greenness → Leisure-time MVPA | .07 (.041) | .06 (.048) | .07 (.041) | .06 (.046) |
| Residential Greenness → Climate Anxiety | –.05 (.181) | –.04 (.214) | –.06 (.128) | –.03 (.291) |
| Nature Connectedness → Climate Anxiety | .10 (.011) | .08 (.024) | .11 (.009) | .09 (.018) |
| Fit Indices | χ^2^/df = 1.95 CFI = .96 TLI = .95 RMSEA = .04 SRMR = .04 | χ^2^/df = 2.02 CFI = .95 TLI = .94 RMSEA = .04 SRMR = .03 | χ^2^/df = 1.91 CFI = .96 TLI = .95 RMSEA = .04 SRMR = .03 | χ^2^/df = 1.99 CFI = .95 TLI = .94 RMSEA = .04 SRMR = .04 |

*Note. N = 918. EVI = Enhanced Vegetation Index; MVPA = moderate-to-vigorous physical activity; NDVI = Normalized Difference Vegetation Index; CFI = Comparative Fit Index; TLI = Tucker–Lewis Index; RMSEA = Root Mean Square Error of Approximation; SRMR = Standardized Root Mean Square Residual. The Primary Model column reproduces the corresponding standardized direct-effect estimates from the final structural model reported in the main manuscript. Campus boundaries were held constant across sensitivity analyses; residential greenness was re-estimated using the specified alternative exposure indicators and buffer sizes. Minor variations in effect magnitudes were observed across alternative exposure specifications; nevertheless, the overall pattern of associations and the substantive interpretation of the findings remained consistent across models.*
